# Incidence and Risk Factors of Early Delirium after Cardiac Surgery

**DOI:** 10.1155/2013/323491

**Published:** 2013-09-12

**Authors:** Ieva Norkienė, Donata Ringaitienė, Vilma Kuzminskaitė, Jūratė Šipylaitė

**Affiliations:** Clinic of Anesthesiology and Intensive Care, Faculty of Medicine, Vilnius University, Siltnamiu 29, 04130 Vilnius, Lithuania

## Abstract

*Introduction*. The aim of our study was to identify the incidence and risk factors of delirium after cardiac surgery implementing Intensive Care Delirium Screening Checklist (ICDSC). *Material and Methods*. 87 patients, undergoing cardiac surgery at Vilnius University hospital, were prospectively monitored for postoperative delirium development, during intensive care unit stay. *Results*. The incidence of postoperative delirium was 13.30%. No statistically relevant preoperative predictors of delirium were found. The duration of surgery was significantly longer in delirium group (4.51 ± 1.15 versus 3.76 ± 0.97 hours, *P* = 0.017). Patients in delirium group more often had blood product transfusions (1.50 (± 1.57) versus 0.49 (± 0.91) *P* = 0.003) and had a higher incidence of low cardiac output syndrome (33.30% versus 3.00%, *P* = 0.004); they were significantly longer mechanically ventilated (24.31 ± 28.35 versus 8.78 ± 4.77 (*P* < 0.001)) hours (OR = 1.15 (1.02–1.28)) and had twice longer ICU stay (5.00 ± 2.22 versus 2.60 ± 1.10 (*P* < 0.001)) days (OR = 1.91 (1.22–3.00)). *Conclusions*. The incidence of delirium after cardiac surgery was 13.3%. Independent predictors of delirium were duration of postoperative mechanical ventilation and intensive care unit stay.

## 1. Introduction

Delirium is the most frequent psychiatric syndrome found in intensive care unit setting. The American Psychiatric Association's Diagnostic and Statistical Manual, 4th edition (DSM-IV), lists four key features that characterize delirium [1]: disturbance of consciousness, a change in cognition, or the development of a perceptual disturbance, with acute onset and fluctuating course with an evidence from the history, physical examination, or laboratory findings that the disturbance is caused by a medical condition, substance intoxication, or medication side effect.

The incidence of postoperative delirium ranges from 10 to 46% in general surgical population and reaches 50–67% among the patients undergoing cardiac surgery [2–4]. Wide variations in reported incidence depend on methodological differences between the studies, mainly concerning the implementation of delirium diagnostic scales and methods [5, 6] and study of population characteristics.

Despite extensive research, acute confusion states after cardiac surgery remain a subject of great importance and controversy. The profound impact of psychotic disturbances on postoperative outcomes was noted in numerous studies. Postoperative delirium has been shown to be associated with prolonged and more costly hospital stay, impaired postoperative cognition, and higher possibility of early postoperative death [7]. Increased incidence of cognitive decline reported after intensive care unit delirium has a major impact on postoperative rehabilitation, social dependency of the patient and overall quality of postoperative life [8]. Monitoring and detection of delirium after surgery remain inconsistent, due to fluctuating course and high prevalence of hypoactive manifestations. In contrast to major neurological complications, postoperative confusion states are less noticed. Moreover, variety of symptoms overlapping with dementia and natural changes of aging brain makes the recognition of this complication extremely difficult in elderly population. Utilization of delirium rating scales and checklists increases the percentage of recognition and ensures a better quality of early treatment. On the other hand, accurate diagnostic tools prevent delirium hyperdiagnostics and treatment administration based on subjective individual perception of patient mental status [8]. 

The aim of our study was to assess the incidence and risk factors for postoperative delirium, implementing the Intensive Care Delirium Screening Checklist (ICDSC) in patients after cardiac surgery at our institution.

## 2. Material and Methods

### 2.1. Study Design

A prospective cohort study was conducted in tertiary referral university hospital between December 2011 and February 2012. Study protocol was approved by Vilnius University Hospital Santariskiu Clinics institutional bioethics committee. All consecutive patients undergoing elective heart surgery were screened for enrolment. Written informed consent was obtained before surgery. 

Patients with central nervous system (CNS), cognitive or mental dysfunction, were excluded from the study. Preoperative cognitive status was evaluated using mini-mental state examination (MMSE) a day before scheduled surgery before anesthesia premedication. Intensive care unit stay less than 24 hours was also an exclusion criterion, in order to eliminate emergence delirium cases.

Preoperative evaluation, premedication, anesthesia, and surgery were performed according to institutional protocols; no adjustments were made for study participants, after the surgery patients were immediately transported to the ICU. At the time of delirium assessment all patients were weaned from mechanical ventilation and able to communicate.

### 2.2. Delirium Screening

Diagnosis of delirium was made using Intensive Care Delirium Screening Checklist (ICDSC). The evaluation is based on 8 fields (1 point each): altered level of consciousness, inattention, disorientation, hallucination-delusion-psychosis, inappropriate speech or mood, psychomotor agitation/retardation, sleep-wake cycle disturbance, and fluctuating course of aforementioned items. An ICDSC score of 4 or greater indicates delirium. Delirium screening was started 24 hours after surgery and repeated every 8 hours during the patients' ICU stay. This study was designated for investigating early onset of delirium following cardiac surgery; therefore screening period was limited to 5 days.

### 2.3. Assessment of Risk Factors

Variables expected to be associated with development of postoperative delirium were divided into 3 categories: preoperative, intraoperative, and postoperative. Information on risk factors was obtained from preoperative interview with the patient and using chart records. The intensity of pain was evaluated using Visual Analogue Scale (VAS).

### 2.4. Statistical Analysis

The incidence of delirium was calculated and compared between coronary artery bypass grafting (CABG) and heart valve surgery patients using Fisher's test. 

Risk factors were analyzed for both groups. Continuous data was presented by mean and standard deviation. Comparison between groups was performed using the Student's *t* test. Categorical variables were presented as proportions and compared using chi-square or Fisher's test where appropriate. All tests were “two-tailed”; *P* value of <0.05 was regarded as statistically significant. Using univariate logistic regression analysis odds ratios with 95% confidence intervals were calculated for each variable. Significant risk factors with narrow confidence intervals were included in multivariate analysis. 

## 3. Results

Final analysis was performed on the data of 87 out of 110 consecutive patients scheduled for heart surgery. 39 patients underwent heart valve surgery (with or without CABG). CABG group consisted of 48 patients. Overall delirium was detected in 12 cardiac surgery patients (13.30%). Hyperactive delirium accounted for only a quarter of cases. In heart valve surgery group the incidence of delirium was 17.95% compared to 8.33% in CABG group. Despite higher incidence of delirium in valve surgery group, the difference was not statistically significant (*P* = 0.209). The results of univariate analysis concerning the influence of studied variables on delirium development are presented in Tables 1, 2, and 3. 

Both delirium and nondelirium groups were similar considering preoperative variables. Neither of preexisting medical conditions, except age more than 75 years, was associated with an increased incidence of postoperative delirium in univariate analysis. 

There were some procedure-related differences between the groups. Patients in delirium group had significantly longer operation and anesthesia times. Use of inotropic support after weaning from CPB had a borderline significance (*P* = 0.057), meanwhile reinstitution of CPB was significantly more often in delirium group (25.0% versus 3%, *P* = 0.02). 

Significant delirium risk factors were associated with postoperative period in the ICU. Patients in delirium group were treated in intensive care twice longer than non delirious patients (OR 2.27, CI = 1.47–3.50). They had prolonged duration of mechanical lung ventilation (OR = 1.18, CI = 1.0–1.31), more positive fluid balance, and increased lactate levels in early postoperative period. Univariate analysis showed a relevant difference in postoperative hemoglobin levels. Patients in delirium group were more often anemic and had higher incidence of blood product transfusions. Multivariate analysis (Table 4) showed that only two variables were independently associated with postoperative delirium development: duration of intensive care unit stay and duration of controlled mechanical ventilation.

## 4. Discussion

Our data confirm the findings of previous studies stating that patients undergoing open heart surgery are in greater risk for developing postoperative delirium than the patients undergoing isolated bypass grafting [9–11]. The incidence of postoperative delirium in our population was twice higher after valve surgery, compared with CABG. However, the overall prevalence of early postoperative delirium, diagnosed using Intensive Care Delirium Screening (ICDS) Checklist, was only 13.3%. We would like to assume that such a low rate of early psychotic disturbances confirms that implementation of accurate delirium screening tool eliminates the chance for delirium hyperdiagnostics in intensive care setting. On the other hand, the similar incidence of postoperative delirium is seen in previous studies using ICDS Checklist for ICU Delirium Screening [12, 13]. Despite extensive research, mechanisms of neuropsychological complications of cardiac surgery remain poorly understood, with some evidence for the contribution of neurotransmission disruption, inflammation, or acute stress responses [13]. Numerous reports suggest that susceptibility for intensive care unit delirium is usually predisposed by a complex interaction of patient-related, environmental, and disease-associated factors [14]. In our study, analysis of nonmodifiable predisposing risk factors for delirium, present on admission to cardiac surgery, did not reveal any significant differences between study groups. Unlike previous authors, we did not find a significant association with a number of factors that we thought would be associated with delirium. Nevertheless these findings were coherent with preoperative comparison of CABG and valve surgery patients, in which it was obvious that both groups were rather similar, concerning potential risk factors for delirium.

On the other hand, analysis of intraoperative variables showed some relevant interactions, with development of postoperative delirium. The importance of cardiopulmonary bypass and cardiac surgery itself in developing postoperative cognitive complications was clearly shown in comprehensive reviews [15]. According to our data intraoperative factors most strongly associated with delirium development were duration of cardiopulmonary bypass time and surgery itself, alongside with fluctuations in hemodynamic after weaning from CPB. These findings suggest that delirium tends to occur more often in patients with more complicated intra-operative course, presumably leading to impaired cerebral perfusion.

Prediction models developed for identification of patients at risk for developing intensive care delirium show a highly prognostic value of factors that are associated with critical illness itself [14, 15]. Moreover in a number of prospective studies it was demonstrated that in the presence of multiple predisposing factors delirium might be easier triggered in patients subjected to intensive care environment [15]. Our results confirm those of previous authors suggesting that intensive care unit stay and the duration of controlled mechanical ventilation were independent predictors of delirium development. Prolonged mechanical lung ventilation also increased the risk for delirium after heart surgery (OR = 1.18, CI = 1.0–1.31). None of the patients had an ICDSC score of at least 4 on their first assessment. This suggests that duration of mechanical lung ventilation could be considered as a risk factor and not a consequence of postoperative delirium.

It was obvious that more complicated postoperative course and low cardiac output syndrome increased vulnerability to psychotic disturbances. Both in valve surgery and CABG patients, blood transfusions appeared to be an independent predictor of delirium development. It is possible to hypothesize that low hemoglobin or anemia could impact neuropsychological performance of the patients, by reducing blood oxygen level in the brain or by lowering a threshold or reserve capacity so that transient ischemia or other factors might have a greater impact on subsequent cognition. However, it is obvious that other comorbidities could prompt both anemia and delirium [16].

Delirium monitoring and timely treatment intervention are a guideline-recommended practice [17]. The Delirium Checklist used in the current investigation is a previously validated instrument for establishing the diagnosis [12, 18]. We succeeded to show that ICDS Checklist, used in post cardiac surgery unit, might be successfully validated and implemented for postoperative delirium screening in our patients. Further data collection and analysis are required for ICDS validation in Lithuanian population. 

Monitoring delirium alone may not be sufficient to change the outcomes. The present investigation was intended as a pilot study, so the major limitation is the small number of patients included. Therefore our findings lack a statistical significance to draw far reaching conclusions. We could only assume that timely correction of modifiable risk factors including impaired intra-operative hemodynamic, anemia, and postoperative mechanical ventilation duration might decrease the incidence of delirium development. 

## 5. Conclusions

The incidence of delirium after cardiac surgery was 13.3%. Postoperative delirium was more frequent in patients with more complicated operative and postoperative course. Independent predictors of delirium in all cardiac surgery population are duration of postoperative mechanical ventilation and intensive care unit stay.

## Figures and Tables

**Table 1:** Univariate analysis of preoperative risk factors for delirium after cardiac surgery.

Variable	D (*N* = 12)	ND (*N* = 67)	*P*	Univariate analysis, OR (95% CI)
Demographic data				
Age (y)	67.67 (±10.46)	64.64 (±10.97)	0.379	1.03 (0.96–1.10)
Age ≥70 years	66.70%	35.80%	0.047	1.05 (0.55–2.04)
BMI (kg/m^2^)	29.06 (±7.76)	27.80 (±4.55)	0.439	1.05 (0.93–1.18)
Daily smokers	8.30%	13.40%	0.625	0.59 (0.07–5.10)
History of alcohol abuse	0%	7.50%	4.429	—
Sleep disturbances	41.70%	40.30%	0.929	1.06 (0.30–3.68)
MMSE (score)	28.00 (±1.49)	27.96 (±1.66)	0.930	0.98 (0.67–1.44)
Operative risk				
EuroScore II (%)	2.36 (±1.41)	1.97 (±1.43)	0.392	0.84 (0.57–1.25)
STS risk score (%)	2.37 (±1.43)	2.18 (±1.31)	0.720	0.90 (0.49–1.67)
Comorbidities				
Diabetes	41.70%	19.40%	0.090	2.97 (0.81–10.86)
Congestive heart failure	91.70%	85.10%	0.472	1.93 (0.22–16.64)
Arterial hypertension	91.70%	89.60%	0.650	1.28 (0.14–11.49)
Atrial fibrillation	25.00%	35.80%	0.354	0.60 (0.15–2.42)
ICA stenosis	8.30%	11.90%	0.588	0.67 (0.08–5.91)
COPD	8.30%	11.90%	0.588	0.67 (0.08–5.91)
Visual impairment	91.70%	88.10%	0.588	1.49 (0.17–13.15)
Hearing impairment	25.00%	50.70%	0.090	0.32 (0.08–1.30)
History of falls	33.30%	25.40%	0.399	1.47 (0.39–5.51)

D: delirium group; ND: nondelirium group; OR: odds ratio; CI: confidence interval; BMI: body mass index; MMSE: Mini-mental state examination; ICA: internal carotid artery; COPD: chronic obstructive pulmonary disease.

**Table 2:** Univariate analysis of intraoperative risk factors for delirium development after cardiac surgery.

Variable	D (*N* = 12)	ND (*N* = 67)	*P*	Univariate analysis, OR (95% CI)
Duration of anesthesia (h)	5.31 (±1.17)	4.51 (±1.04)	0.019	1.18 (1.06–2.97)
Duration of surgery (h)	4.52 (±1.15)	3.76 (±0.97)	0.017	1.82 (1.02–3.12)
Duration of CPB (min)	132.67 (±39.05)	119.46 (±44.45)	0.346	0.99 (0.98–1.01)
Aortic cross-clamp time (min)	60.06 (±18.03)	75.98 (±30.90)	0.484	0.99 (0.97–1.01)
Minimal temperature (°C)	29.75 (±2.92)	31.31 (±3.07)	0.107	1.00 (0.99–1.00)
MAP (mm Hg)	58.07 (±13.94)	63.11 (±9.79)	0.129	1.04 (0.99–1.10)
Lactate levels (mmol/L)	1.78 (±0.79)	1.31 (±0.61)	0.023	2.50 (1.07–5.84)
Reinstitution of CPB	25.00%	3.00%	0.023	3.00 (2.59–73.91)
Inotropes after CPB	97.40%	51.70%	0.053	6.14 (0.75–50.50)

D: delirium group; ND: non-delirium group; OR: odds ratio; CI: confidence interval; CPB: cardiopulmonary bypass; MAP: mean arterial pressure; SaO_2_: arterial oxygen saturation; SBE: standard base excess.

**Table 3:** Univariate analysis of postoperative risk factors for delirium development after cardiac surgery.

Variable (mean (±SD), or %)	D (*N* = 12)	ND (*N* = 67)	*P*	Univariate analysis, OR (95% CI)
ICU stay (days)	5.00 (±22)	2.60 (±1.10)	0.000	2.27 (1.47–3.50)
Duration of CMV (h)	24.31 (±28.35)	8.78 (±4.77)	0.000	1.18 (1.0–1.31)
Fluid balance (L)	4.83 (±2.21)	3.57 (±1.89)	0.048	1.00 (1.00–1.00)
Drainage (L)	0.61 (±0.52)	0.49 (±0.28)	0.250	0.99 (0.99–1.001)
Pain intensity (VAS)	3.75 (±2.42)	3.28 (±2.21)	0.509	0.91 (0.69–1.20)
Hb (g/L)	90.50 (±13.37)	100.99 (±15.27)	0.029	0.95 (0.91–0.99)
Hct (%)	28.45 (±4.03)	32.85 (±4.63)	0.054	1.15 (0.99–1.33)
Lactate levels >2.5 mmol/L	75.00%	34.30%	0.010	0.17 (0.04–0.71)
RBC transfusions (unts)	1.50 (±1.57)	0.49 (±0.91)	0.003	1.99 (1.20–3.31)
FFP transfusions (unts)	0.92 (±2.39)	0.07 (±0.61)	0.014	1.58 (0.95–2.62)
Use of inotropes	100%	77.60%	0.064	3.73 (—)
Resternotomy	16.70%	1.50%	0.059	13.20 (1.09–159.35)
Rhythm/conduction disorders	58.30%	22.90%	0.055	3.29 (0.93–11.61)
Low cardiac output	33.30%	3.00%	0.004	16.25 (2.56–103.30)
CNS events	8.30%	1.50%	—	—

D: delirium; ND: non-delirium; OR: odds ratio; CI: confidence interval; ICU: intensive care unit; CMV: controlled mechanical ventilation; VAS: visual analogue scale; Hb: hemoglobin; Hct: hematocrit; RBC: red blood cells; FFP: fresh-frozen plasma; IABC: intraaortic balloon counterpulsation; CNS: central nervous system.

**Table 4:** Multivariate analysis of risk factors associated with delirium development after cardiac surgery.

Variable	Odds ratio	95% Confidence interval
Intensive care unit stay (days)	1.91	1.22–3.00
Duration of controlled mechanical ventilation	1.15	1.02–1.28
